# Targeting GPRC5D With CAR‐T Cells in Relapse/Refractory Multiple Myeloma: Case Report and Literature Review

**DOI:** 10.1155/crh/5900306

**Published:** 2025-12-15

**Authors:** Sijia Yan, Xi Ming, Jiaying Wu, Dan Peng, Mi Zhou, Yi Xiao

**Affiliations:** ^1^ Department of Hematology, Tongji Hospital, Tongji Medical College, Huazhong University of Science and Technology, Wuhan, Hubei, China, hust.edu.cn; ^2^ Department of Nuclear Medicine, Tongji Hospital, Tongji Medical College, Huazhong University of Science and Technology, Wuhan, China, hust.edu.cn

**Keywords:** chimeric antigen receptor T cell, G-protein-coupled receptor class C group 5 member D, multiple myeloma, relapsed/refractory

## Abstract

Multiple myeloma (MM) is the second most common hematologic malignancy, and patients with relapsed/refractory MM (RRMM) face limited treatment options and a poor prognosis. Recently, CAR‐T cell therapy targeting G‐protein‐coupled receptor, class C group 5 member D (GPRC5D) has shown promising efficacy and safety in preclinical studies, offering new hope for patients with RRMM. We report the successful treatment of a 48‐year‐old female patient with relapsed/refractory nonsecretory MM. The patient had high‐risk factors, including 1q21 amplification and TP53 deletion, and had relapsed after seven lines of therapy, including autologous hematopoietic stem cell transplantation, proteasome inhibitors, immunomodulatory agents, PD‐1 inhibitors, and CD38 monoclonal antibodies. She also developed extramedullary disease. Eventually, she received CAR‐T cell therapy targeting GPRC5D, which led to the complete disappearance of extramedullary lesions and a sustained complete remission lasting up to 17 months. In conclusion, CAR‐T cell therapy targeting GPRC5D is highly effective and well‐tolerated in patients with RRMM, especially those with high‐risk factors. Further studies with larger cohorts and longer follow‐up periods are needed to validate the clinical application of GPRC5D‐targeted CAR‐T cell therapy in RRMM, particularly for patients who have failed BCMA‐targeted therapies.

## 1. Introduction

Multiple myeloma is the second most prevalent hematologic malignancy, characterized by increasing incidence and prevalence, imposing a substantial global health burden [1]. Despite the availability of comprehensive treatment strategies for both newly diagnosed and relapsed patients, most individuals inevitably face disease recurrence posttherapy. Furthermore, a subset of patients advances to a refractory state, leaving them with limited therapeutic options and a bleak prognosis after undergoing multiple treatment regimens [2]. Proteasome inhibitors, such as carfilzomib and ixazomib, combined with immunomodulatory agents like lenalidomide, have become the standard‐of‐care regimens for relapsed and refractory myeloma. Targeted and immunotherapeutic agents, including daratumumab (a CD38 monoclonal antibody) and elranatamab (a B‐cell mature antigen [BCMA]×CD3 bispecific antibody), have shown remarkable efficacy in patients with relapsed and refractory multiple myeloma (RRMM), significantly enhancing overall response rates (ORRs) and progression‐free survival (PFS) [3, 4]. Additionally, chimeric antigen receptor T (CAR‐T) cell therapies targeting BCMA have become a viable option for patients with relapsed and refractory diseases, with four products now approved for clinical use. However, approximately 45% of patients treated with BCMA CAR‐T cells experience relapse after achieving initial remission [5], underscoring the urgent need to identify novel therapeutic targets.

G‐protein‐coupled receptor, class C group 5 member D (GPRC5D), an orphan G‐protein‐coupled receptor, is highly expressed on the surface of tumor cells in over 50% of patients with myeloma [6]. The existing preclinical studies and clinical trial results collectively suggest that GPRC5D‐targeted CAR‐T cells offer significant potential for patients with multiple myeloma, particularly those who relapse following anti‐BCMA treatment. In our case report, we successfully treated a patient with relapsed and refractory nonsecretory multiple myeloma, who had multiple high‐risk factors, including extramedullary disease and TP53 deletion, using GPRC5D‐targeted CAR‐T cells. This treatment resulted in the disappearance of extramedullary disease and achieved a complete remission lasting for 17 months without the need for other antimyeloma therapies.

### 1.1. Case Presentation

A 48‐year‐old female patient presented with unexplained rib pain in June 2015. A whole‐body bone scan showed abnormal uptake of bone tracer, indicative of multiple myeloma. Bone marrow biopsy revealed active marrow proliferation with 89% plasma cells. Fluorescence in situ hybridization (FISH) detected amplification signals at the 1q21 locus (abnormal cell ratio, 5%) and deletion of the TP53 signal. Immunoglobulin heavy chain gene (IGH) locus showed signals of translocation and rearrangement, with an abnormal cell ratio of 60.5%. Immunofixation electrophoresis of serum and urine did not find monoclonal immunoglobulin. Based on these findings, she was diagnosed with nonsecretory multiple myeloma, revised international staging system (R‐ISS) Stage III, characterized by 1q21 amplification and TP53 deletion. The patient had undergone multiple treatment regimens, including autologous hematopoietic stem cell transplantation, proteasome inhibitors, immunomodulatory agents, PD‐1 inhibitors, and CD38 monoclonal antibodies. Despite these treatments, she relapsed with extramedullary disease.

**Figure 1:**
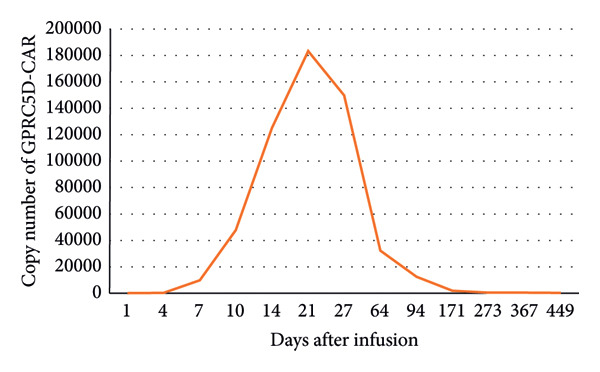
Trend of GPRC5D‐CAR during follow‐up.

On July 7, 2023, the patient underwent T‐cell collection in the preparation for GPRC5D‐targeted CAR‐T cell therapy and started bridge chemotherapy, which included daratumumab 600 mg on Days 1–3 and mitoxantrone liposome 40 mg on Day 1. On August 8, 2023, the patient received an infusion of GPRC5D‐targeted CAR‐T cells at a dose of 3.0 × 10^6^/kg. Postinfusion, CAR‐T cells expanded rapidly in the patient’s body, reaching a peak GPRC5D‐CAR copy number around 1 month later (Figure 1).

**Figure 2:**
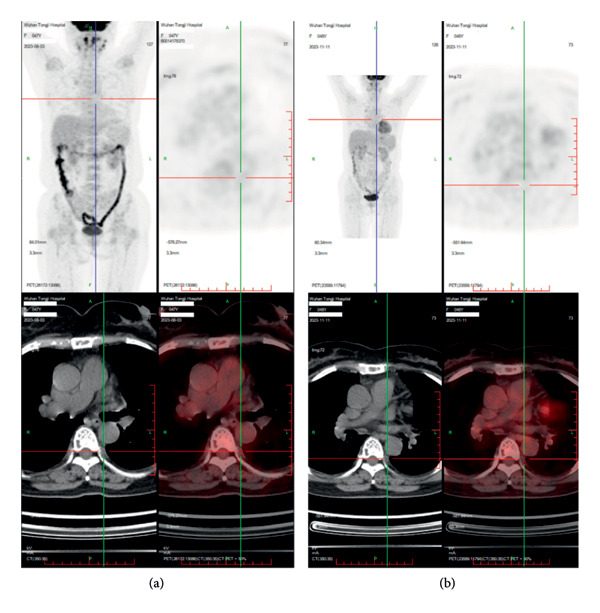
Comparison of extramedullary lesions on PET‐CT in the patient before and after treatment. Prior to GPRC5D‐CAR‐T therapy, the patient exhibited a soft‐tissue density lesion measuring approximately 12 mm × 4 mm adjacent to the thoracic vertebrae (a), which was suggestive of an extramedullary lesion. However, 3 months following treatment, the soft tissue density lesion had completely resolved (b).

The patient experienced Grade 2 cytokine release syndrome (CRS), characterized by fever, nausea, general fatigue, and hypotension. Symptomatic management led to a complete recovery without the need for glucocorticoids. Follow‐up bone marrow aspirations, performed on November 10, 2023, January 25, 2024, May 7, 2024, August 8, 2024, October 29, 2024, and January 15, 2025, showed sustained stringent complete response (sCR) with no detectable residual disease on cytology and flow cytometry. Imaging studies consistently showed the disappearance of the soft‐tissue density adjacent to the thoracic vertebrae, indicating resolution of extramedullary disease (Figure 2).

## 2. Discussion

Multiple myeloma has seen significant advancements in both diagnostic and therapeutic strategies. Despite these improvements, some patients continue to experience cycles of relapse and remission, ultimately developing RRMM. For these patients, who have relapsed after multiple lines of treatment, the options for effective therapeutic agents remain limited. The rapid evolution of cellular immunotherapy, particularly CAR‐T cell therapy, has introduced new hope for patients with hematologic malignancies. Currently, four CAR‐T cell therapies—Abecma, Carvykti, CT103A, and zevor‐cel—have been approved for the treatment of RRMM in China. All these therapies target BCMA and have demonstrated robust safety and efficacy profiles in patients with relapsed and refractory myeloma. However, a subset of patients still experiences relapse following BCMA‐targeted therapies [5], underscoring the urgent need to develop novel therapeutic targets.

GPRC5D, an orphan G‐protein‐coupled receptor, is highly expressed on the surface of tumor cells in over 50% of patients with myeloma. Its expression in normal tissues is restricted to the hair follicles of the skin, the filiform papilla epithelial cells of the tongue, and hard keratinized tissues [7]. This unique expression pattern makes GPRC5D a promising therapeutic target for myeloma. Preclinical studies have demonstrated that GPRC5D CAR‐T cells can effectively eliminate myeloma cells, including those resistant to BCMA‐targeted therapies, in xenografted mouse models. Clinical trials have shown that various GPRC5D‐targeted CAR‐T cell therapies have been effectively used to treat RRMM, demonstrating favorable efficacy and safety profiles. These CAR‐T cells exhibited long‐term persistence without inducing severe toxicity or adverse reactions [8] (Table 1).

**Table 1:** GPRC5D CAR‐T cell therapy in patients with RRMM.

CAR‐T	N	ORR (%)	CRR (%)	CRS (%)	ICANS (%)	Previous anti‐BCMA treatment (%)
MCARH109 [9, 10]	17	71	35	88	6	70
OriCAR‐017 [11]	10	100	100	100	0	50
BMS‐986393 [12]	70	86	38	43	11	75
Xia et al. [13]	37	84	35	70	2	100
Li et al. [14]	7	85.7	42.8	85.7	0	28.6
CT071 [15]	20	100	50	60	5	25

Abbreviations: CAR‐T, chimeric antigen receptor T cell; ORR, overall response rate; CRR, complete response rate; CRS, cytokine release syndrome; ICANS, immune effector cell‐associated neurotoxicity syndrome; BCMA, B‐cell mature antigen.

In our study, the patient exhibited multiple high‐risk factors, including TP53 deletion, 1q21 amplification, and extramedullary disease. Despite undergoing seven lines of therapy, including hematopoietic stem cell transplantation, proteasome inhibitors, PD‐1 inhibitors, and CD38 monoclonal antibodies, the patient relapsed. During the patient’s visit to our hospital, a relapse with extramedullary disease was confirmed. Antigen assessment indicated a high positivity rate of 97.6% for GPRC5D in the tumor cells. Consequently, we administered GPRC5D‐targeted CAR‐T cell therapy. Posttreatment, the extramedullary disease resolved completely. The patient did not undergo maintenance therapy for multiple myeloma following the CAR‐T cell treatment and achieved complete remission lasting for 17 months.

However, because GPRC5D is also found on keratin‐producing cells, GPRC5D‐targeted CAR‐T cells may cause unique off‐tumor toxicities. Previous studies frequently reported nail toxicity; for instance, in the Phase I trial of MCARH109, 65% of patients experienced Grade 1 nail toxicity [9]. In the OriCAR‐017 study, 30% of participants suffered from nail loss [11], and in research by Xia et al., 24% encountered nail changes [13]. Fortunately, these nail toxicities were self‐limiting and resolved without intervention. Skin toxicities, such as rashes and dryness, were effectively managed with topical corticosteroids. Taste disturbances also improved on their own. Additionally, GPRC5D is expressed at low levels in the inferior olivary nucleus of the medulla, which is involved in transmitting motor and sensory signals to the cerebellum and regulating motor coordination [16]. In Mailankody et al.’s study, two patients developed cerebellar toxicity, exhibiting symptoms of cerebellar dysfunction like gait instability, ataxia, and dysarthria, while cognitive, motor, and tactile functions were unaffected. These patients received treatments including oral corticosteroids, high‐dose methylprednisolone, intravenous immunoglobulin, and methotrexate, which stabilized the disease. At the last follow‐up, their symptoms remained stable but persistent [9]. Similarly, in Bal et al.’s research, two patients treated with BMS‐986393 also experienced cerebellar toxicity [12]. Thus, cerebellar dysfunction might be a characteristic off‐tumor toxicity associated with GPRC5D‐targeted therapy. However, the limited number of patients treated with these CAR‐T cells means there is a lack of large‐scale clinical data to draw conclusions about its incidence and management strategies. In our case, the patient experienced only Grade 2 CRS and transient bone marrow suppression lasting 1 week, without any ICANS or off‐tumor toxicities, including nail changes. The small sample size in our study could explain the absence of severe adverse reactions, indicating that further expansion is necessary to validate these findings.

Similar to BCMA‐targeted CAR‐T cell therapy, the risk of relapse persists after GPRC5D‐targeted CAR‐T cell treatment. In the follow‐up of patients treated with MCARH109, six patients experienced relapse between 3 and 9 months posttreatment. Immunohistochemistry and polymerase chain reaction analyses indicated that all relapsed patients showed reduced or absent GPRC5D mRNA and protein expression at the time of disease progression [17]. Consequently, antigen escape is likely the primary cause of these relapses. Ma et al. performed whole‐genome sequencing on samples from 10 patients who relapsed after GPRC5D CAR‐T cell therapy and found that 8 cases had GPRC5D loss, while 2 cases exhibited mixed expression (GPRC5D±). In myeloma cell lines, GPRC5D expression is inversely correlated with the methylation level of its regulatory region. In five samples after treatment, there were multiple hypermethylated sites in the transcriptional regulatory elements of the GPRC5D gene. These results suggest that biallelic inactivation and epigenetic silencing driven by hypermethylation are key mechanisms leading to GPRC5D antigen escape and treatment resistance [18]. To address the critical issues of heterogeneous expression of target antigens on tumor cells and the downregulation of these antigens during treatment, current research has developed a dual‐specificity CAR‐T cell incorporating both anti‐BCMA and anti‐GPRC5D single‐chain variable fragments. This dual‐specificity CAR‐T cell can recognize both antigens simultaneously, maintaining cytotoxic activity even when the expression level of one antigen decreases. Currently, BCMA/GPRC5D dual‐specific CAR‐T cells have been used to treat patients with RRMM, showing favorable safety and efficacy profiles [19]. Yao et al. used BCMA/GPRC5D bispecific CAR‐T cells to treat nine RRMM patients with EMD. All patients achieved PR or better, with 44.4% achieving CR. The median follow‐up was 6.08 months. The 1‐year OS and PFS were 60% and 63%, respectively, and the median OS and PFS had not been reached. CRS occurred in 66.7% of patients, all of which were Grade 1–2, and ICANS was not observed [20]. Dual‐targeting CAR‐T cell therapy reduces the risk of antigen escape and has preliminarily demonstrated feasibility and safety in RRMM. However, longer follow‐up and larger sample size studies are still needed for validation.

## 3. Conclusion

In conclusion, multiple myeloma, a relatively common hematologic malignancy, is well managed with established treatment protocols. For patients with relapsed or refractory disease after multiple lines of therapy, CAR‐T cell therapy has proven to be an effective option. Currently, CAR‐T cells targeting BCMA are widely used in clinical practice and have reached a relatively advanced stage of development. GPRC5D‐targeted CAR‐T cell therapy has shown significant potential for treating patients with RRMM, particularly those with high‐risk factors such as TP53 deletion and 1q21 amplification. Our case report demonstrates the complete disappearance of extramedullary lesions and a sustained complete remission lasting up to 17 months. Further studies with larger cohorts and longer follow‐up periods are needed to validate the clinical application of GPRC5D‐targeted CAR‐T cell therapy in RRMM, particularly for patients who have failed BCMA‐targeted therapies.

**Nomenclature**
BCMA: B cell mature antigen
CAR‐T: Chimeric antigen receptor T cell
CCDN1: Cyclin D1 gene
CRR: Complete response rate
CRS: Cytokine release syndrome
CT: Computed tomography
EMD: Extramedullary disease
FISH: Fluorescence in situ hybridization
GPRC5D: G‐protein‐coupled receptor, class C group 5 member D
ICANS: Immune effector cell‐associated neurotoxicity syndrome
IGH: Immunoglobulin heavy chain gene
MM: Multiple myeloma
ORR: Overall response rate
OS: Overall survival
PACE: Cisplatin, doxorubicin, cyclophosphamide, and etoposide
R‐ISS: Revised international staging system

## Consent

The participant provided her written informed consent to participate in this study. Written informed consent was obtained from the individual for the publication of any potentially identifiable images or data included in this article.

## Disclosure

All authors have read and approved the final manuscript.

## Conflicts of Interest

The authors declare no conflicts of interest.

## Author Contributions

S.Y. and X.M. were major contributors to the writing of the manuscript. J.W. was responsible for the revision of the article. D.P. conducts a detailed analysis of the results of imaging examinations. Y.X. and M.Z. supervised the writing of the article and provided the critical review. S.Y. and X.M. contributed equally to the study.

## Funding

This review was supported by the National Natural Science Foundation of China (No. 81873444, No. 82070213, and No. 82370196 to Yi Xiao).

## Data Availability

The data that support the findings of this study are available from the corresponding author upon reasonable request.
